# The burden of respiratory syncytial virus (RSV) associated acute lower respiratory infections in children with Down syndrome: A systematic review and meta–analysis

**DOI:** 10.7189/jogh.07.020413

**Published:** 2017-12

**Authors:** Markus Chan, John J Park, Ting Shi, Federico Martinón–Torres, Louis Bont, Harish Nair, Respiratory Syncytial Virus Network (ReSViNET)

**Affiliations:** 1 Usher Institute of Population Health Sciences and Informatics, University of Edinburgh, UK; 2 Harvard TH Chan School of Public Health, Harvard University, Boston, Massachusetts, USA; 3 Department of Public Health and Primary Care, Leiden University Medical Centre, Leiden, the Netherlands; 4 Translational Pediatrics and Infectious Diseases, Pediatrics Department, Hospital Clínico Universitario de Santiago de Compostela, Santiago de Compostela, Spain; 5 Wilhelmina Children's Hospital, University Medical Center Utrecht, Utrecht, Netherlands; * Joint first authroship

## Abstract

**Background:**

Acute lower respiratory tract infections (ALRIs) caused by respiratory syncytial virus (RSV) are a leading cause of hospitalization in infants. Numerous risk factors have been identified in the aetiology of severe RSV–associated ALRI necessitating hospitalisation, including prematurity and congenital heart disease. Down syndrome (DS), a common genetic disorder associated with congenital and dysmorphic features, has recently been identified as an independent risk factor for RSV–associated ALRI requiring hospitalisation; however, the disease burden of RSV–associated ALRI in this population has not yet been established. Similarly, the impact of DS as an independent risk factor has not yet been quantified. We aimed therefore to estimate the incidence of admissions in children with DS, and by comparing this with unaffected children, to quantify the risk of DS independent of other risk factors.

**Methods:**

A systematic review of the existing literature published between 1995 and March 1, 2017 was performed to quantify the incidence of hospitalisation due to RSV–associated ALRI in children with DS. Meta–analyses were performed on extracted data using STATA statistical software, and hospitalisation rates for children with and without DS under the age of 2 were calculated.

**Findings:**

5 articles were ultimately deemed eligible for analyses. Analyses were limited to children under the age of 2 years. We calculated the hospitalisation rate for children with DS in this age group to be 117.6 per 1000 child–years (95% CI 67.4–205.2), vs a rate of 15.2 per 1000 child–years (95% CI 8.3–27.6) in unaffected children. This indicates DS contributes to a 6.8 (95% CI 5.5–8.4) fold increase in the relative risk of hospitalisation for RSV–associated ALRI.

**Interpretation:**

Though limited by a small number of articles, this review found sufficient evidence to conclude DS was a significant independent risk factor for the development of severe RSV–associated ALRI requiring hospitalisation. Further studies are needed to define the impact of DS in conjunction with other comorbidities on the risk of severe RSV infection. Determining benefits of immunoprophylaxis or future vaccines against RSV in this at–risk population is warranted.

Respiratory syncytial virus (RSV) is one of the leading causes of acute lower respiratory tract infections (ALRIs) in young children (<5 years) globally [1–3].

Almost every child will have been infected with RSV by the age of 2 years, and 40% of these will develop ALRIs. Of these, 0.5–2% of children will require hospitalisation, making RSV–associated ALRIs the leading cause of hospitalisation among infants during winter [1]. Prematurity, chronic lung disease, age <6 months, and congenital heart disease (CHD) are established risk factors for RSV infection [4–6]. Bloemers et al. highlighted Down syndrome (DS) as an independent risk factor for RSV infection [7].

DS is the single most common chromosomal abnormality among live born infants, characterised by congenital and dysmorphic features [8]. Individuals with DS are more prone to numerous health issues, and the condition is associated with an overall increased risk of mortality [9,10]. Notably, ALRIs are the most common cause of hospitalisation among children with DS [11]. However, the burden of RSV–associated ALRI in children with DS is unclear. Further investigation into the epidemiology of RSV infection in paediatric DS populations may help optimise patient care in this vulnerable group.

We aimed to estimate the disease burden of RSV–associated ALRI in children with DS, focussing on hospitalisation and severity of disease. We also aimed to quantify the effect of DS as a dependent and independent risk factor for hospitalisation with RSV–associated ALRI.

## METHODS

### Search strategy and selection criteria

We conducted a systematic literature review using a combination of search terms, hand searching of online journals, and scanning of reference lists of identified citations. Two authors (JP and MC) searched the following databases – MEDLINE, Embase, Global Health, CINAHL, LILACS, Web of Science, IndMED, WHOLIS, and SIGLE. Both extracted data independently.

We included primary, population–based studies published between 1995 and March 1, 2017 reporting hospitalisation rates of RSV–associated ALRI in populations with DS over at least one (1) RSV season. Articles not available in English, and articles using an unclear or inconsistent case definition were excluded. Studies satisfying these minimum eligibility criteria were included. The full eligibility criteria are summarised in **Table 1**.

**Table 1:** Inclusion and exclusion criteria

**Inclusion criteria**	Primary population–based studies reporting incidence data for RSV ALRI requiring hospitalisation
	Articles with a surveillance period of at least 1 year or 1 RSV season
	Articles reporting case fatality ratios of RSV infection
	Articles assessing the OR or RR for children with DS for at least 1 year
	Articles confirming RSV infection by laboratory diagnosis or hospital discharge records of confirmed RSV
	Articles reporting estimates for children separately by immunoprophylaxis status, if included in the study
**Exclusion criteria**	All non–primary articles, including reviews, presentations
	Articles not available in English
	Articles not investigating RSV infection as a primary outcome
	Articles using a case definition of influenza or influenza–like illness
	Articles using an unclear or inconsistent case definition

RSV – respiratory syncytial virus, ALRI – acute lower respiratory tract infection, OR – odds ratio, RR – relative risk, DS – Down syndrome

The review was also registered on the PROSPERO database (Record #59594). Full details of the search strategy used may be found on the PROSPERO database, and in **Online Supplementary Document(Online Supplementary Document)**.

### Definitions

ALRI has been defined as equivalent to clinical pneumonia, which is characterised by acute–onset cough or respiratory distress with age–adjusted tachypnoea. Down syndrome was defined as equivalent to having trisomy 21 based on medical or census records. RSV–associated ALRI was defined as ALRI in a child who is RSV positive (either based on laboratory confirmation or relevant ICD codes in medical records).

To increase the data available for analysis, methodology for confirming RSV infection or coding were not used as eligibility criteria, with the understanding that this could limit the interpretation of the results. We similarly did not distinguish between first or subsequent hospitalisations.

### Data extraction

Hospitalisation rates for all populations and subgroups from relevant studies were extracted onto a Microsoft Excel 2013 database. Information regarding study characteristics, diagnostic tests used, and population characteristics was recorded. Outcome data and risk factor effect parameters were extracted if present. Relative risk and hospitalisation rate data were calculated using raw input data as well as reported figures in the reviewed studies.

### Data analysis

We conducted meta–analyses of the data using STATA version 11.2 (StataCorp LP, College Station TX, USA). Primary outcomes were hospitalisation rates of RSV–associated ALRI in children with DS or without DS, and the impact of DS on hospitalisation rate expressed as relative risk. Pooled estimates with corresponding 95% CIs were reported based on random effects model (DerSimonian–Laird method) since significant heterogeneity was expected across studies [12,13].

Two meta–analyses were performed: one using hospitalisation rate data, and one with relative risk data. Sensitivity analyses by age were planned, but were not performed due to limited data.

## RESULTS

**Figure 1:**
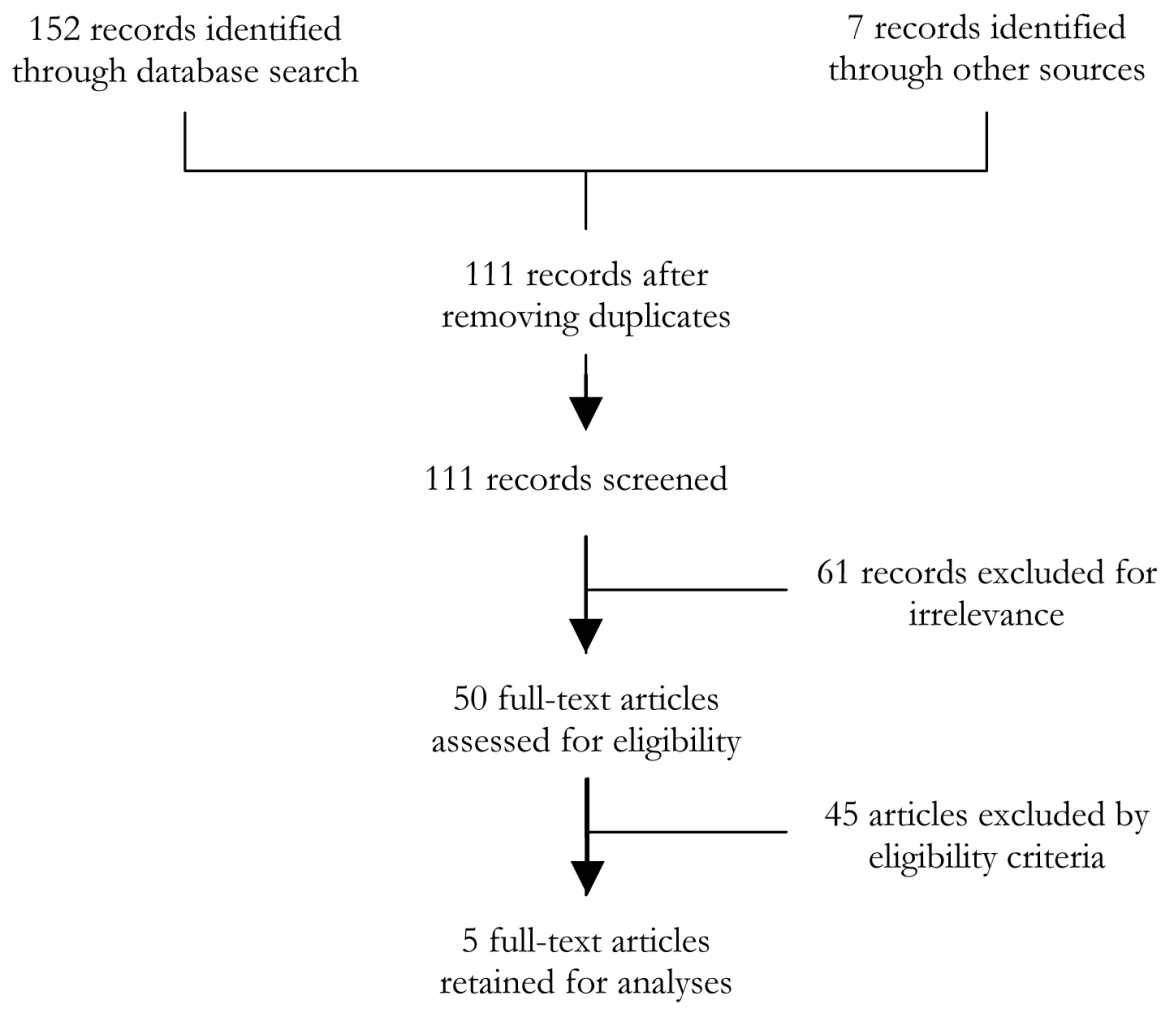
PRISMA flow diagram.

The literature search identified 111 records for screening after excluding duplicates. Of these, 50 articles were reviewed fully, and 5 articles were ultimately identified as meeting our strict eligibility criteria [7,14–17]. This is shown in **Figure 1**.

All 5 studies used passive hospital data and 1 used both active and passive methods of data collection. Study populations were under the age of 2 years [7,14,15,17], except one study which included infants up to 36 months [16]. All studies used hospital in–patient or out–patient data, and did not distinguish between first or repeat hospitalisation episodes. All studies were based in high–income countries: 3 studies were from Europe and 2 were from the USA. No studies in Asia, Africa or South America met eligibility criteria. These articles are summarised in **Table 2** [7,14–17].

**Table 2:** Summary of articles in review

DS – patients with Down syndrome, Non–DS – patients without Down syndrome, RR – relative risk, OR – odds ratio, LoS – length of stay

Data analyses focused on the <2 years old age group, given the paucity of data beyond this group. The implications of this decision are discussed below.

### Hospitalisation rate

Four of the 5 studies were included in the meta–analysis of hospitalisation rates for children under the age of 2 years with and without DS. The meta–analysis found the hospitalisation rate of RSV–associated ALRI in children under the age of 2 years with DS was 117.6 (95% CI 67.5–205.2) per 1000 children per year. Comparatively, the hospitalisation rate of RSV–associated ALRI in children under the age of 2 years without DS was 15.2 (95% CI 8.3–27.6) per 1000 children per year, (*P* < 0.01).

The meta–estimate of relative risk of DS for hospitalisation with RSV–associated ALRI (including data from all 5 studies) in this age group was 6.8 (95% CI 5.5–8.4).

**Figure 2:**
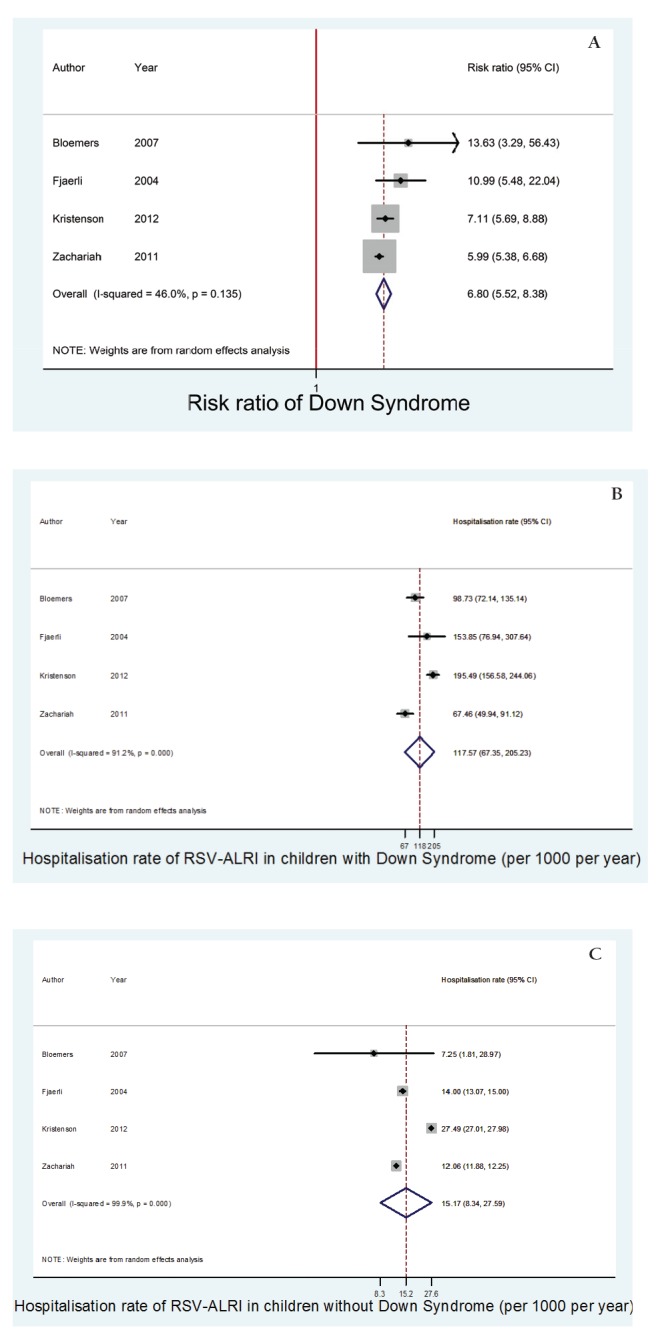
Forest plots of (a) risk ratio of Down syndrome for respiratory syncytial virus (RSV)–associated ALRI requiring hospitalisation in children under the age of 2 years; (b) hospitalisation rate of RSV–associated acute lower respiratory tract infection (ALRI) in children under the age of 2 years with Down syndrome; and (c) hospitalisation rate of RSV–associated ALRI in children under the age of 2 years without Down syndrome.

These findings are summarised in **Table 3** and **Figure 2**.

**Table 3:** Summary of articles, with meta–analyses and sensitivity analyses

Author	RSV–associated ALRI hospitalisation rate among patients with DS, per 1000 child–years (95% CI)	RSV–associated ALRI hospitalisation rate among patients without DS, per 1000 child–years (95% CI)	RR (95% CI)
Bloemers et al. [7]	98.7 (72.1–135.1)	7.3 (1.8–29.0)	13.6 (3.3–56.4)
Fjaerli et al. [14]	153.9 (76.9–307.6)	14.0 (13.1–15.0)	11.0 (5.5–22.0)
Kristensen et al. [15]	195.5 (156.6–244.1)	27.5 (27.0–28.0)	7.1 (5.7–8.9)
Stagliano et al. [16]	96.2 (77.4–119.6)	14.2 (13.9–14.5)	6.8 (5.5–8.4)
Zachariah et al. [17]	67.5 (49.9–91.1)	12.1 (11.9–12.3)	6.0 (5.4–6.7)
**Meta–analysis of all articles:**			
Meta–analysis for all under 2 years*	117.6 (67.4–205.2)	15.2 (8.3–2.6)	6.8 (5.5–8.4)

RSV – respiratory syncytial virus, ALRI – acute lower respiratory tract infection, RR – relative risk, CI – confidence interval

*Sensitivity analysis using data from Bloemers et al. [7], Fjaerli et al. [14], Kristensen et al. [15], and Zachariah et al. [17] only.

### Disease severity data

Four of the 5 studies compared disease severity in children with and without DS ; however, this was inconsistent. Distinct criteria were used across the studies, including need for respiratory support [16,17], need for mechanical ventilation [16,17], median duration of hospital stay [14,16,17], and mean duration of stay [15].

The wide range of severity measures precluded formal analyses of the data; however, within the group of hospitalised children, RSV–associated ALRI was consistently more severe in children with DS compared to those without DS [14–17]. This is shown in **Table 2**.

### Comorbidities

Three of the 5 studies investigated the role of comorbid risk factors in the epidemiology of RSV–associated ALRI in children with DS. However, there were inconsistencies in the conditions assessed, and the parameters used.

Only two studies formally assessed the impact of comorbid risk factors; however, analyses were limited to comparing populations with DS and any additional risk factors to populations with DS without any risk factors [7,17]. The inconsistencies between the studies precluded formal meta–analyses of the data. Both studies found the presence of additional risk factors generally did not significantly affect the hospitalisation rate, clinical presentation, or management of RSV–associated ALRI in children with DS [7,17]. However, Zachariah et al. noted a significantly higher use of bronchodilators in populations with DS and at least one additional risk factor compared to populations with DS alone [17].

Importantly, all three studies found an increased hospitalisation rate for RSV–associated ALRI in populations with DS without additional risk factors [7,16,17].

## DISCUSSION

To our knowledge, this is the first systematic review and meta–analysis to report global estimates for hospitalisation rates attributable to RSV–associated ALRI in children with DS compared to children without DS. This is important since RSV remains a leading cause of ALRIs and hospitalisation among children globally [1,2]. We estimated children under the age of 2 years with DS had a 6.8 times (95% CI 5.5–8.4) increased risk of hospitalisation for RSV–associated ALRI compared to children in the same age group without DS. This suggests DS is an important risk factor for RSV–associated ALRI hospitalisation and comparable to other risk factors like CHD (RR 1.5–2.7) [18–21] and bronchopulmonary dysplasia (RR 10.7) [21].

Though meta–analyses were not performed on severity measures, studies assessing infection severity consistently found more severe RSV–associated ALRI in hospitalized children with DS compared to those without DS. Generally, a higher proportion of children with DS required respiratory support and required longer hospitalisation [14–17].

### Down syndrome and RSV infection

Several proposed mechanisms explain the increased susceptibility of children with DS to RSV–associated ALRI. These children are more likely to have airway abnormalities such as poor upper airway tone and abnormal lung development. These may contribute towards a tendency to airway compromise and oedema, and thus severe ALRIs [15,22,23]. Children with DS have also been shown to have high rates of CHD and pulmonary hypertension, both of which are risk factors for severe RSV–associated ALRI [8,20]. While these comorbidities are independent risk factors for the development of severe RSV–associated ALRI, the impact of these risk factors in conjunction with DS remains unclear.

Immunological dysfunction may also have a role: DS has been associated with poor thymus development and lower numbers of B and T cells, which may contribute to prolonged infections and poor microbial clearance [24–27].

Lastly, the presence of DS may be associated with a lower threshold for admission, given the complex care needs which may be present in affected infants. This is particularly true in the first year of life, where there is generally poorer nutritional intake [16,28]. However, this does not fully explain the increased risk suffered by children with DS, and disease severity likely also has a role [7].

### Comorbidities and Down syndrome

Only three studies reported hospitalisation rate data in populations with DS and other comorbidities, limiting formal analyses on the role of comorbid risk factors alongside DS in the epidemiology of RSV–associated ALRI [7,16,17].

Children with DS often have other risk factors for severe RSV–associated ALRI. Notably, CHD, a significant risk factor for severe RSV–associated ALRI, is comorbid in up to 55% of children with DS [29,30]. However, the reviewed studies consistently showed children with DS alone remained at significantly greater risk of severe RSV–associated ALRI necessitating hospitalisation [7,16,17].

This review suggests there are limited studies exploring the impact of DS in conjunction with other risk factors in the epidemiology of RSV–associated ALRI. Our preliminary findings suggest children with DS and other risk factors are at similar risk of severe RSV–associated ALRI compared to children with DS alone, but further research is necessary [7,16,17].

### Palivizumab prophylaxis for children with Down syndrome

Palivizumab is a monoclonal IgG–1 antibody which has been shown to reduce the incidence of RSV hospitalisation, particularly in high–risk groups [31]. However, due to the high costs associated with it, licensing has largely been limited to high–risk populations such as infants with haemodynamically significant CHD (HSCHD) [32].

This systematic review suggests DS alone is associated with a significantly increased risk of hospitalisation due to RSV–associated ALRI. Furthermore, our results suggest that DS alone is associated with more severe RSV infection. While a meta–analysis could not be performed, the consistency of the findings is striking. However, there are other factors to consider when discussing the benefit of immunoprophylaxis in this population.

There are currently no large randomised–control trials investigating the efficacy of immunoprophylaxis for the prevention of RSV–associated ALRI specifically in children with DS. Yi et al. found a lower rate of RSV–related hospitalisation in infants with DS who were treated with palivizumab compared to a retrospective group of untreated controls [33]. A recent observational study by Mikami et al. investigated the efficacy of palivizumab in preventing RSV–associated hospitalisations in immunocompromised populations, including children with DS. However, the study was uncontrolled, and data for children with DS were not explicitly available. Further studies are therefore required to determine the efficacy of palivizumab in patients with DS.

Currently, guidance for palivizumab variably includes a statement on DS. Where this is explicitly mentioned, the guidance further varies, from not routinely recommended to reasonably recommended [33,34]. Many groups cite uncertainty over the burden of disease and the efficacy of palivizumab immunoprophylaxis in this population as challenges limiting the guidance [33–35].

Despite being at increased risk, no studies investigating RSV vaccination or new immunoprophylactic agents to our knowledge have included children with DS in their study populations. Similarly, further information on the topic will be similarly needed to guide RSV prevention in children with DS. Notably, studies on safety and cost–effectiveness in this at–risk population would be helpful in establishing guidance on this issue.

### Study limitations

The main limitation in this study was the small number of articles eligible for analysis. This reflects the paucity of research in this area, and demonstrates the need for further investigation into the epidemiology of RSV–associated ALRIs in populations with DS. Notably, several studies pooled populations with and without immunoprophylaxis, and were therefore excluded from the meta–analyses.

Similarly, data were limited regarding the role of comorbidities and other confounding factors such as CHD and immunodeficiency. Only three studies assessed the role of DS in addition to other risk factors. While all three studies found DS to be an independent risk factor for RSV–associated hospitalization, the role of DS in addition to other risk factors in the epidemiology of RSV–associated ALRI is likely complex and currently not clearly understood [7,15].

In particular, the excess risk carried by DS in conjunction with major risk factors remains unknown, and clarification in this area would help define the vulnerability of this at–risk population.

Inconsistencies between the studies reviewed also limited the internal validity of this study. Due to limited data, analyses were restricted to children under the age of 2 years. While RSV–associated ALRI primarily affects younger infants (<2 years), severe infection has also been reported in older children (<5 years) [2,16]. This may be especially true for children with DS: this review found children with DS were older at admission than control populations, though this was statistically insignificant [14,16,17]. However, at least one study has shown children with DS to be significantly older than unaffected children at time of hospitalisation [36].

Similarly, the reporting of comorbidities and disease severity, where available, was variable. Notably, studies assessing the impact of risk factors did so by the general presence or absence of risk factors, limiting comparisons.

The precise definition of RSV–associated ALRI varied between studies, though they were broadly similar. Differences arose primarily in case ascertainment, with some studies relying on laboratory–confirmed RSV status [7,14] and others using hospital discharge coding [15-17]. Due to paucity of studies, we did not analyse data by method of case ascertainment. Notably, this review is thus subject to the limitations of the studies included, particularly regarding the accuracy of ICD coding.

Reporting overall hospitalisation rates may over–represent more vulnerable individuals, resulting in inaccurate data. This effect may be exaggerated when considering some guidelines advocate admitting children for social or geographic reasons [37,38]. Risk factors for repeated admission may be distinct from risk factors of severe infection, and these nuances may be lost when measuring hospitalisation rates alone.

A greater limitation in the application of this review is the scope of the review, and the socio–geographical bias of the eligible articles. Only five studies were retained for the meta–analyses, and all were based either in Europe or North America. Notably, no articles from low– and middle–income countries (LMICs) fulfilled eligibility criteria, though previous reviews have highlighted the significant disease burden of RSV–associated ALRI in such countries [2,39]. While DS may reasonably be assumed to be a risk factor everywhere, the burden of disease in these populations may vary depending on local public health programmes.

Another limitation of this review was the age of the studies included in the review. Much of the data used in the meta–analyses came from before 2005. As population health and the efficacy of medical interventions have improved in this time, and continue to do so, it is possible the data do not reflect current trends in RSV–associated ALRI hospitalisation and standards of hospital care; this is especially true in high income settings.

Guidelines for palivizumab immunoprophylaxis have also changed during this time, notably for children with HSCHD [33,40]. The treatment of HSCHD has similarly improved in recent years [41]. Theoretically, correcting HSCHD should reduce the risk of developing RSV–associated ALRI, though there are yet limited data to support this hypothesis. These developments could also affect the data in this review.

### Future research

The meta–analysis identified a significant need for good data on the impact of RSV–associated ALRI in populations with DS. Notably, further research into the impact of DS on the epidemiology of RSV–associated ALRI in combination with other risk factors would help clarify the burden of disease in this population. Similarly, research investigating the risk carried by populations with DS over time could give insight into the pathophysiology underlying this relationship. This would also help define the risk of children with DS to develop repeat episodes of RSV–associated ALRI.

Importantly, all the studies included in this review came from high–income countries in Europe or the Americas. Data from other geographic regions, particularly in LMICs, would help identify regional variations, and may help establish guidance for RSV prevention globally.

Similarly, to assess better the role of immunoprophylaxis, studies investigating the efficacy and tolerability of palivizumab specifically in infants with DS are needed. Cost–effectiveness analyses in this population may also be helpful, though these analyses may be limited by geographic variation in the costs and benefit of immunoprophylaxis. Furthermore, as palivizumab immunoprophylaxis becomes introduced more widely, the effects of changing policies on the epidemiology of RSV–associated ALRI in this population should be explored.

## CONCLUSION

This is the first systematic review investigating the burden of RSV–associated ALRI in children with Down syndrome. Our findings suggest this population is at significantly greater risk of severe RSV–associated ALRI than unaffected populations, and may benefit from being considered for immunoprophylaxis. Unfortunately, the efficacy of palivizumab in this population is still unclear, and warrants further research, including cost–effectiveness analysis. When vaccines or extended half–life antibodies against RSV will become available, their efficacy should be explored in this vulnerable population.

## 

**Table 2:** Summary of articles in review

Authors	Study setting	Study period	Population	Hospitalisation rate (per 1000 child–years)	OR/RR of hospitalisation (DS vs non–DS)	Severity measurement
Bloemers et al. [7]	Amsterdam and Leiden, Netherlands	1976 – 2005	<36 mo; 395 DS; 276 non–DS; Not receiving palivizumab	DS: 98.7 (72.1–135.1)	13.6 (3.3–56.4)	
				Non–DS: 7.3 (1.8–29.0)		
Fjaerli et al. [14]	Akershus, Norway	1993 – 2000	<2 y; 7 DS; 70 other non–DS high–risk; 687 non–DS; Low–risk	DS: 153.9 (76.9–307.6)	11.0 (5.5–22.0)	Median LoS; DS: 7.5 d; Non–DS: 4 d; (*P* < 0.001)
				Non–DS: 14.0 (13.1–15.0)		
Kristensen et al. [15]	Denmark	1997 – 2003	0 – 23 mo;399 DS; 391 584 non–DS	DS: 195.5 (156.6–244.0)	7.1 (5.7–8.9)	Mean LoS; DS: 1.91 d; Non–DS: 1.0 d; (*P* < 0.001)
				Non–DS: 27.5 (27.0–28.0)		
Stagliano et al. [16]	USA	2005 – 2011	<36 mo; 842 DS; 632 358 non–DS	DS: 96.2 (77.4–119.6)	6.8 (5.5–8.4)	Need for respiratory support; DS: 9.3%; Non–DS: 1.8%; (*P* < 0.001); Median LoS; DS: 4 d; Non–DS: 2 d;(*P* < 0.01)
				Non–DS: 14.2 (13.9–14.5)		
Zachariah et al. [17]	Colorado, USA	1995 – 2006	0 – 23 mo; 630 DS;	DS: 67.5 (49.9–91.1)	6.0 (5.4–6.7)	Median LoS; DS: 4–5 d; Non–DS: 2–3 d; (*P* < 0.001)
				Non–DS: 12.1 (11.9–12.3)		

DS – patients with Down syndrome, Non–DS – patients without Down syndrome, RR – relative risk, OR – odds ratio, LoS – length of stay, d – day, mo – month, y – year
