# Supplementary material for: The burden of respiratory syncytial virus (RSV) associated acute lower respiratory infections in children with Down syndrome: A systematic review and meta–analysis
Source: J Glob Health. 2017 Dec 4;7(2):020413. doi: 10.7189/jogh.07.020413 (PMC5735780; doi:10.7189/jogh.07.020413)
Supplement: Online Supplementary Document [file jogh-07-020413-s001.pdf]

Online Supplementary Document

Chan et al. The burden of respiratory syncytial virus (RSV) associated acute lower respiratory infections in children with Down syndrome: A systematic review and meta–analysis

J Glob Health 2017;7:020413

SEARCH STRATEGY BY DATABASE

Ovid MEDLINE(R) – 1946 to February Week 3 2017

1. exp Respiratory Syncytial Viruses/ or exp Respiratory Syncytial Virus, Human/ or exp Respiratory Syncytial Virus Infections/ or RSV.mp.
2. respiratory syncytial virus*.mp.
3. pneumonia.mp. or exp Pneumonia/ or exp Pneumonia, Viral/
4. bronchiolitis.mp. or exp Bronchiolitis/ or exp Bronchiolitis, Viral/
5. exp Respiratory Tract Infections/ or respiratory infection*.mp.
6. exp Respiratory Tract Diseases/ or respiratory disease*.mp.
7. incidence.mp. or exp Incidence/
8. prevalence.mp. or exp Prevalence/
9. exp Child Mortality/ or exp Infant Mortality/ or mortality.mp. or exp Hospital Mortality/ or exp Mortality/
10. death*.mp. or exp Death/ or exp "Cause of Death"/
11. morbidity.mp. or exp Morbidity/
12. burden.mp.
13. epidemiology.mp. or exp Epidemiology/
14. Down Syndrome.mp. or exp Down Syndrome/
15. Down’s syndrome.mp.
16. Trisomy 21.mp. or exp trisomy 21/
17. 1 or 2
18. 3 or 4 or 5 or 6
19. 7 or 8 or 9 or 10 or 11 or 12 or 13
20. 14 or 15 or 16
21. 18 or 19
22. 17 and 20 and 21
23. limit 22 to yr="1995 -Current"

Embase – 1980 to 2017 Week 09

1. exp Respiratory syncytial pneumovirus/ or exp respiratory syncytial virus infection/

2. rsv.mp.

3. respiratory syncytial virus*.mp.

4. bronchiolitis.mp. or exp bronchiolitis/

5. pneumonia.mp. or exp pneumonia/

6. exp respiratory tract infection/ or respiratory infection*.mp.

7. exp respiratory tract disease/ or respiratory disease*.mp.

8. exp incidence/ or incidence.mp.

9. prevalence.mp. or exp prevalence/

10. mortality.mp. or exp mortality/

11. exp death/ or death*.mp.

12. morbidity.mp. or exp morbidity/

13. burden.mp.

14. exp epidemiology/ or epidemiology.mp.

15. (down syndrome or down?s syndrome).mp. or exp Down syndrome/

16. trisomy 21.mp. or exp trisomy 21/

17. 1 or 2 or 3

18. 4 or 5 or 6 or 7

19. 8 or 9 or 10 or 11 or 12 or 13 or 14

20. 15 or 16

21. 18 or 19

22. 17 and 20 and 21

23. limit 22 to yr="1995 -Current"

Global Health – 1910 to 2017 Week 07

1. exp human respiratory syncytial virus/

2. rsv.mp.

3. respiratory syncytial virus*.mp.

4. pneumonia*.mp. or exp pneumonia/ or exp community acquired pneumonia/

5. bronchiolitis.mp. or exp bronchiolitis/

6. exp respiratory diseases/

7. exp lower respiratory tract infections/

8. respiratory infection*.mp.

9. respiratory disease*.mp.

10. incidence.mp. or exp incidence/

11. prevalence*.mp. or exp disease prevalence/

12. mortality.mp. or exp mortality/

13. death*.mp. or exp death/ or exp "causes of death"/

14. morbidity.mp. or exp morbidity/

15. exp epidemiology/ or epidemiology.mp.

16. burden.mp.

17. (down syndrome or down?s syndrome).mp. or exp down's syndrome/

18. trisomy 21.mp.

19. 1 or 2 or 3

20. 4 or 5 or 6 or 7 or 8 or 9

21. 10 or 11 or 12 or 13 or 14 or 15 or 16

22. 17 or 18

23. 20 or 21

24. 19 and 22 and 23

25. limit 24 to yr="1995 -Current"

CINAHL

S1= (MH “respiratory syncytial virus infections”) OR (MH “respiratory syncytial viruses”) OR “respiratory syncytial virus”

S2= “RSV”

S3= (MH “pneumonia+”) OR “pneumonia” OR (MH “pneumonia, viral”) OR (MH “community-acquired pneumonia”)

S4= (MH “bronchiolitis+”) OR “bronchiolitis”

S5= (MH “respiratory tract infections+”) OR “respiratory infection”

S6= (MH “respiratory tract diseases+”) OR “respiratory disease”

S7= (MH “incidence”) OR “incidence”

S8= (MH “prevalence”) OR “prevalence”

S9= (MH “mortality+”) OR “mortality” OR (MH “infant mortality”) OR (MH “child mortality”) OR (MH “hospital mortality”)

S10= (MH “death+”) OR “death” OR (MH “cause of death”) OR (MH “infant death+”)

S11= (MH “morbidity+”) OR “morbidity”

S12= “burden”

S13= (MH “epidemiology+”) OR “epidemiology”

S14= (MM "Down Syndrome") OR "down syndrome"

S15= “trisomy 21”

S16= “downs syndrome”

S17= S1 OR S2

S18= S3 OR S4 OR S5 OR S6

S19= S7 OR S8 OR S9 OR S10 OR S11 OR S12 OR S13

S20= S14 OR S15 OR S16

S21= S18 OR S19

S22= S17 AND S20 AND S21

Limiters: Published date: 19950101 –

Web of Science

You searched for: TITLE: (respiratory syncytial virus OR RSV) AND TITLE: (trisomy 21 OR down syndrome OR downs syndrome) AND TOPIC: (epidemiology OR prevalence OR incidence) Timespan: 1995-2017. Indexes: SCI-EXPANDED, SSCI, A&HCI, CPCI-S, CPCI-SSH, BKCI-S, BKCI-SSH, ESCI, CCR-EXPANDED, IC.

WHOLIS (KMS)

words or phrase "respiratory syncytial virus" AND subject "epidemiology" AND words or phrase "down syndrome"

LILACS

(tw:(respiratory syncytial virus)) AND (tw:(epidemiology)) AND (tw:(down syndrome))

IndMED

Respiratory syncytial virus OR rsv

AND epidemiology OR prevalence OR incidence

AND down syndrome OR downs syndrome OR trisomy 21

- Restrict to medIND

SIGLE

respiratory syncytial virus AND down syndrome

Section/topic	#	Checklist item	Reported on page #
**TITLE**			
Title	1	Identify the report as a systematic review, meta-analysis, or both.	1
**ABSTRACT**			
Structured summary	2	Provide a structured summary including, as applicable: background; objectives; data sources; study eligibility criteria, participants, and interventions; study appraisal and synthesis methods; results; limitations; conclusions and implications of key findings; systematic review registration number.	2
**INTRODUCTION**			
Rationale	3	Describe the rationale for the review in the context of what is already known.	3
Objectives	4	Provide an explicit statement of questions being addressed with reference to participants, interventions, comparisons, outcomes, and study design (PICOS).	3
**METHODS**			
Protocol and registration	5	Indicate if a review protocol exists, if and where it can be accessed (e.g., Web address), and, if available, provide registration information including registration number.	4
Eligibility criteria	6	Specify study characteristics (e.g., PICOS, length of follow-up) and report characteristics (e.g., years considered, language, publication status) used as criteria for eligibility, giving rationale.	4, 17
Information sources	7	Describe all information sources (e.g., databases with dates of coverage, contact with study authors to identify additional studies) in the search and date last searched.	4
Search	8	Present full electronic search strategy for at least one database, including any limits used, such that it could be repeated.	22-26
Study selection	9	State the process for selecting studies (i.e., screening, eligibility, included in systematic review, and, if applicable, included in the meta-analysis).	4, 17
Data collection process	10	Describe method of data extraction from reports (e.g., piloted forms, independently, in duplicate) and any processes for obtaining and confirming data from investigators.	4
Data items	11	List and define all variables for which data were sought (e.g., PICOS, funding sources) and any assumptions and simplifications made.	4
Risk of bias in individual studies	12	Describe methods used for assessing risk of bias of individual studies (including specification of whether this was done at the study or outcome level), and how this information is to be used in any data synthesis.	X

Summary measures	13	State the principal summary measures (e.g., risk ratio, difference in means).	4
Synthesis of results	14	Describe the methods of handling data and combining results of studies, if done, including measures of consistency (e.g., I^2) for each meta-analysis.	5

Section/topic	#	Checklist item	Reported on page #
Risk of bias across studies	15	Specify any assessment of risk of bias that may affect the cumulative evidence (e.g., publication bias, selective reporting within studies).	X
Additional analyses	16	Describe methods of additional analyses (e.g., sensitivity or subgroup analyses, meta-regression), if done, indicating which were pre-specified.	5
**RESULTS**			
Study selection	17	Give numbers of studies screened, assessed for eligibility, and included in the review, with reasons for exclusions at each stage, ideally with a flow diagram.	6, 19
Study characteristics	18	For each study, present characteristics for which data were extracted (e.g., study size, PICOS, follow-up period) and provide the citations.	6, 17-18
Risk of bias within studies	19	Present data on risk of bias of each study and, if available, any outcome level assessment (see item 12).	X
Results of individual studies	20	For all outcomes considered (benefits or harms), present, for each study: (a) simple summary data for each intervention group (b) effect estimates and confidence intervals, ideally with a forest plot.	17-18
Synthesis of results	21	Present results of each meta-analysis done, including confidence intervals and measures of consistency.	6-7, 18, 20-21
Risk of bias across studies	22	Present results of any assessment of risk of bias across studies (see Item 15).	X
Additional analysis	23	Give results of additional analyses, if done (e.g., sensitivity or subgroup analyses, meta-regression [see Item 16]).	6-7, 18, 20-21
**DISCUSSION**			
Summary of evidence	24	Summarize the main findings including the strength of evidence for each main outcome; consider their relevance to key groups (e.g., healthcare providers, users, and policy makers).	8
Limitations	25	Discuss limitations at study and outcome level (e.g., risk of bias), and at review-level (e.g., incomplete retrieval of identified research, reporting bias).	10-11

Conclusions	26	Provide a general interpretation of the results in the context of other evidence, and implications for future research.	11-12
**FUNDING**			
Funding	27	Describe sources of funding for the systematic review and other support (e.g., supply of data); role of funders for the systematic review.	13

From: Moher D, Liberati A, Tetzlaff J, Altman DG, The PRISMA Group (2009). Preferred Reporting Items for Systematic Reviews and Meta-Analyses: The PRISMA Statement. PLoS Med 6(7): e1000097. doi:10.1371/journal.pmed1000097
